# Illegal waste fly-tipping in the Covid-19 pandemic: enhanced compliance, temporal displacement, and urban–rural variation

**DOI:** 10.1186/s40163-022-00170-3

**Published:** 2022-09-25

**Authors:** Anthony C. Dixon, Graham Farrell, Nick Tilley

**Affiliations:** 1 School of Law, University of Leeds, Leeds, UK; 2 University College London, London, UK

## Abstract

**Objective:**

Illegal dumping of household and business waste, known as fly-tipping in the UK, is a significant environmental crime. News agencies reported major increases early in the COVID-19 pandemic when waste disposal services were closed or disrupted. This study examines the effect of lockdowns on illegal dumping in the UK.

**Method:**

A freedom of information request was sent to all local authorities in the UK asking for records of reported incidents of fly-tipping for before and after the first national lockdown. ARIMA modelling and year-on-year comparison was used to compare observed and expected levels of fly-tipping. Urban and rural local authorities were compared.

**Results:**

A statistically significant decline in fly-tipping during the first lockdown was followed by a similar increase when lockdown ended. The effects largely cancelled each other out. There was pronounced variation in urban–rural experience: urban areas, with higher rates generally, experienced most of the initial drop in fly-tipping while some rural authorities experienced an increase.

**Conclusion:**

Waste services promote compliance with laws against illegal dumping. When those services were disrupted during lockdown it was expected that fly-tipping would increase but, counter-intuitively, it declined. This enhanced compliance effect was likely due to increased perceived risk in densely populated urban areas. However, as lockdown restrictions were eased, fly-tipping increased to clear the backlog, indicating temporal displacement.

**Supplementary Information:**

The online version contains supplementary material available at 10.1186/s40163-022-00170-3.

## Introduction

Huge increases in illegal waste dumping, known as fly-tipping in the UK (British Broadcasting Corporation [BBC], 2017), were claimed early in the pandemic when waste services were closed or disrupted during a national lockdown:

“*Britons dump tons of rubbish, clothes ‘recycling’ on the street in coronavirus lockdown clear-outs*” (Gallagher, in the Daily Mail, 2 April 2020)

“*Fly-tipping is up 300 per cent during coronavirus lockdown after closure of council tips and charity shops.”* (Edmunds, in the Daily Mail, 30 April 2020)

The BBC reported that one city

“*has seen hundreds of cases of fly-tipping in the past month, with people being tempted to dispose of their waste illegally during the lockdown*”

and that

“*A fly-tipping reporting page told [the BBC that] cases uploaded to its site across the UK were up 75%.”* (BBC, 2020).

Local news and other agencies reported similar experiences elsewhere, some emphasising rural problems (Newton, 2020; Darlington & Stockton Times, 2020; Countryside Alliance, 2020). One professional source claimed that closure of recycling centres would “inevitably drive even greater criminality in the waste sector” (Circular for Resource and Waste Professionals, 2020). These sources informed preliminary academic work (Roberts et al., 2020; Tilley, 2020).

A more recent survey of local authority workers, sponsored by the Department for the Environment, Food and Rural Affairs (DEFRA), the responsible government body, reported that:

“*There was also a perceived increase in smaller fly-tipping incidents after April 2020 in places that did not normally see much fly-tipping. Stakeholders considered this was perpetrated by less organised people, probably local householders.*

*…Three quarter of respondents (76%) believed the fly-tipping situation in their local area had got worse since the start of the COVID-19 pandemic, including 34% who described it as ‘a lot worse’.*

*…Some [local authority workers] considered that waste infrastructure challenges during the COVID-19 pandemic have induced behaviour changes, resulting in more fly-tipping. It was for example thought by some LAs that the pandemic had spawned a new group of people who had the intention to dispose of waste the right way, but fly-tipped after becoming frustrated by various obstacles (e.g. a HWRC being closed).”* (Purdy, 2022, 18–19)

With the exception of the last one which was more recent, these sources informed the identification of our preliminary research questions: Was there an increase in fly-tipping early in the pandemic? Was it a national problem, or particular to rural areas? More broadly, what was the extent, nature and variation in fly-tipping in the pandemic? The DEFRA-sponsored study published in 2022 highlights the policy-relevance, the pervasiveness, and durability of the issues addressed here.

The environmental crime of fly-tipping imposes significant costs on society (Smith, 2020). The harms caused depend on the type of waste being dumped and the location. For example, toxic materials dumped in watercourses produce health hazards and harms to the environment. Building materials dumped on private land incur removal costs for landowners. In the UK, local authorities bear the costs of clearing material dumped on publicly owned land. Unsightly illegal dumping in public view comprises a public nuisance. A study by the waste collection and recycling industry body, the Environmental Services Association, estimated that the economic costs of fly-tipping in England increased from £604 million in 2015 to £924 million in 2018/19 (ESA, 2021). Annual national statistics from DEFRA show that in 2018/19, the latest year for which official data prior to the pandemic was available, over a million incidents were recorded (1,072,431), with London accounting for around a third (Department for the Environment, Food and Rural Affairs [DEFRA], 2019).

The growing body of research into crime in the pandemic spans many countries (e.g. Borrion et al., 2020 on China; Estévez-Soto, 2021 on Mexico; Halford et al., 2020 and Langton et al., 2020, 2021 on the UK; Payne et al., 2021 on Australia; Nivette et al., 2021 on global effects). However, there appears to be little empirical research into the effects on waste crime. The present study relates to waste from households and businesses in the UK. The terms illegal waste disposal, illegal dumping, and fly-tipping are used interchangeably. Clinical waste (Agamuthu & Barasarathi, 2021; Brown & Gilliam, 2020; Lan et al., 2021) is not addressed here.^1^

Publicly available data on fly-tipping was not appropriate for use in this study. DEFRA makes quarterly fly-tipping data publicly available. However, quarterly data means seasonal effects confuse interpretation of trends. It also means that analysis of events—whether national, regional, or local—is impossible because dates are imprecise. This meant it was not feasible to link pandemic-specific dates and events to the quarterly public data. The absence of location information also means identification of hotspots is not possible, while waste crime on private land is largely overlooked. There is little incentive for fly-tipping on private land to be reported because its removal is the responsibility of landowners.

To overcome data limitations, a novel survey of illegal waste was undertaken for this present study. The survey is described after details of waste management in the UK and the theoretical underpinnings of the study are given.

### Waste management in the UK

Waste management is heavily regulated across the UK, with responsibility for regulation and provision of services devolved to local authorities, except for major incidents that are handled by national environment agencies for England, Scotland, Wales and Northern Ireland. A primary reference here is the governmental department with responsibility for national policy and oversight of waste management for England, which is the Department for Environment, Food and Rural Affairs (DEFRA). The regulatory responsibilities of local authorities and environment agencies are sometimes supported by other enforcement bodies, notably local police and the National Crime Agency.

Local government authorities across the UK are responsible for the regular collection of routine household waste, generally on a weekly or fortnightly basis.^2^ It is funded from taxation, and otherwise free to householders at the point of delivery. There are separate receptacles and collections for general waste and that for recycling. Many, though not all, authorities also provide separate bins for food waste. ‘Green waste’ that is produced seasonally during the growing season is generally collected separately and charged to householders who opt into the service. Local authority household waste collection is mostly from individual addresses, although apartment blocks may have collective receptacles.

Household Waste Recycling Centres (HWRCs) where bulky and excess waste materials can be deposited lawfully by householders, are also operated by local authorities. Businesses that collect, transport and dispose of waste materials are obliged to have licenses to do so. All producers of waste have a ‘duty of care’ to ensure that the waste materials they produce are disposed of legally, including large-scale, commercial waste producers.

The pandemic brought dramatic change, including to waste services. On March 23rd 2020, the first national stay-at-home lockdown was announced. The lockdown prescribed that people could leave their homes only for specific purposes: for designated essential work, to purchase essentials, for medical purposes, or to exercise (for an hour at most). Shops selling non-essential goods, hotels, hostels, gyms, libraries, places of worship, and campsites were closed. Effectively all social events (involving more than six household members) were banned, and individuals were advised to remain 2m apart to avoid transmission of the virus. Relaxation of the initial lockdown rules began on May 11th 2020, with further liftings in early June then early July, although precautionary behaviours were still advised (see Barber et al., 2021 for more detailed description of legislative change).

With respect to waste management, DEFRA issued eight rounds of guidance to Local Authorities in England between April 8th 2020 and December 14th 2020, to deal with changing levels of infection, developing understanding of COVID-19, the initial lockdown and alterations in government measures to attempt to control the pandemic (DEFRA, 2020). These guidance documents, since withdrawn, were largely advisory. Guidance was particularly needed to assist local authorities in determining which services were deemed essential. The guidance urged councils to prioritise food waste and black bin collection, focusing on perishable household waste. Weekly collection of recycling material was suspended. The basis for the guidance was to accord with the national lockdown but also to accommodate staff shortages due to infection or self-isolation (required when an individual had been in contact with someone infected).

The vast majority of local authorities closed HWRCs because they were deemed non-essential: 77 percent of these services were withdrawn and a further 21 percent severely disrupted in the week beginning 30 March, 2020 (Association of Directors of Environment, Economy, Planning and Transport, the Local Authority Recycling Advisory Committee, the National Association of Waste Disposal Officers and the Local Government Association [ADEPT], 2020). In effect, nearly all council tips, as they are colloquially known, were not open for business as usual. Details of commercial waste centres are not known to the research team. By May 11th, however, waste collection services and HWRCs were beginning to revert to previous patterns of provision, and most had resumed by June.

Non-HWRC waste collection services were less affected overall, as shown in Table 1 for the week beginning 30 March, 2020. Garden waste and bulk waste disposal experienced the most significant disruption with large proportions of their services withdrawn.

**Table 1:** Percent of local authorities and disruption in waste collection by type of service during initial Covid-19 lockdown (Week of 30 March, 2020). Source: ADEPT (2020)

	Type of waste collection					
	Recycling (%)	Garden (%)	Food (%)	Bulky (%)	Clinical (%)	Residual
Operating normally	73	41	63	19	88	83%
Minor disruption	20	12	18	12	9	15%
Moderate disruption	4	8	8	3	3	2%
Severely disrupted	2	1	1	3	0	0
Services withdrawn	1	38	9	65	0	0

‘Residual’ waste is that which is not covered in the other categories

### Theoretical framework

This study is underpinned by routine activity and rational choice theories and the applied framework of situational crime prevention (Eck & Clarke, 2019; Felson & Clarke, 1998). Patterns of everyday life generate situations where potential offenders interact with suitable targets in the absence of capable intermediaries.^3^ In the context of the present study, then, patterns of waste crime reflect variation in the opportunity for its commission across time and place. Most offending, including the decision to dump waste illegally, reflects the ‘bounded rationality’ of offenders: offences occur when and where the balance of risk, effort and reward mean breaking the law seems beneficial. Situational crime prevention seeks to increase costs or reduce the benefits to offending. It seeks to change situations and systems, including management systems, in ways that make crime less attractive or tempting. It suggests that, in the present context, illegal waste dumping is undertaken largely because it is easier, cheaper, more convenient or more rewarding than legal disposal (Hodsman & Williams, 2011). Waste services, in turn, help reduce waste crime by facilitating compliance with the law. Previous research from the perspective of situational prevention emphasised the need to focus on hotspot times and places where fly-tipping is concentrated (Webb et al., 2006). This does not mean that preventing fly-tipping is easy or straightforward. An evaluation of ten ‘clean-up’ operations, where fly-tip waste was removed, found that, contrary to expectation, they encouraged fly-tipping. The study concluded that this was because offenders learned they could offend with impunity and that somebody else would clean-up their mess (Brown & Evans, 2012).

Criticisms of situational crime prevention have been largely rebutted (Clarke & Bowers, 2017; Farrell & Tilley, 2020). Most relevant here is the criticism that crime is displaced rather than prevented. The best evidence finds displacement often does not occur or is typically less than complete. Partial displacement is thus a signature of effective prevention, but also indicates that crime can be strategically deflected to less harmful forms (Barr & Pease, 1990). Moreover, a diffusion of crime prevention benefits, where preventive effects spread beyond the intended scope, is at least as common (Guerette & Bowers, 2009; Johnson et al., 2014).

The literature on crime in the pandemic, mentioned earlier, describes how the pandemic has changed routine activities in ways that affect crime. Reduced movement of people occurred in many areas of life, including on public transport and in the workplace, in and around retail, entertainment and leisure locations, and elsewhere. This decreased the number of potential interactions of potential offenders and potential targets (whether people or other types of target) for direct contact predatory crimes such as personal robbery and theft, which would incur greater risk and effort. In other instances, the mechanism by which crime opportunities were reduced was different: stay-at-home requirements increased home guardianship and surveillance and led to reduced burglary and local vehicle theft (Halford et al., 2020). The opportunities for some online crimes, however, increased due to increased online household activities. Similar, crimes occurring within the household, including domestic violence and child abuse, increased in some instances due to increased interaction of potential offenders and suitable targets at home (Kourti et al., 2021).

With respect to fly-tipping, the pandemic might change the crime opportunity structure via various routes. The media sources quoted in the introduction suggested that the pandemic had increased fly-tipping. Waste disposal services facilitate compliance with the law, and their removal might be expected to cause increased fly-tipping. Reduced scope for legal disposal at HWRCs plus disruptions to routine waste collection, could generate increased supply of material for illegal dumping. In addition, guardianship of potential fly-tipping sites could be reduced in lockdown. The actual and perceived risk of breaking lockdown laws might also vary between areas, perhaps reflecting population density and natural surveillance levels in urban and rural areas. Some pandemic-related changes might be expected to reduce fly-tipping. Other things equal, if everyone adhered to stay-at-home rules, then fly-tipping would decline. Of course, the illegal nature of fly-tipping could suggest that offenders might ignore stay-at-home rules, and while the highly novel pandemic situation makes this uncertain, it is clear that there were significant numbers of breaches of lockdown rules (Halford et al., 2022). This raises the issue of the different populations of potential offenders: might people who previously did not fly-tip begin fly-tipping in the pandemic? Would more habitual and prolific fly-tippers change their output? What would be the balance between these possibilities. Overall, then, these different possibilities mean that the aggregate expected effect on fly-tipping is uncertain. However, drawing on the limited evidence from news and other sources plus the assessment of theory outlined above, our initial expectation and hypothesis was that fly-tipping would be found to have increased during the first national lockdown.

## Methods

### Data

Local authorities in England are obliged to keep records of fly-tipping incidents and to return quarterly counts to DEFRA. The returns give overall counts of incidents, counts of types of waste fly-tipped, and the approximate volumes of goods fly-tipped. However, testing the hypothesis outlined required data for shorter time periods because lockdown policies did not align with calendar quarters.

‘Freedom of Information’ requests were sent by email to all relevant local authorities in the UK. For present purposes these were dealt with as Environmental Information Requests or ‘EIRs’. As local authorities are public authorities, they are obliged to provide information requested within 20 days of receipt of a request.^4^ There are exceptions to the obligation, typically when there is an unreasonable cost in collating the data or when the data is too sensitive for release.

The following information was requested: (1) Monthly counts of fly-tips; (2) Monthly counts of fly-tipping by primary waste type; (3) Monthly counts of fly-tipping by land type; and (4) Monthly counts of fly-tipping by waste/incident size. Each local authority was asked for data from 1 January 2017 to 31 July 2020.

Monthly returns were received from 55 percent (216 of 394) of local authorities. We undertook analytic checks to verify the representativeness of the sample. To do this we aggregated the data and compared it to the population of data in the annual national DEFRA dataset for the period during which the two overlapped (DEFRA, 2019). The historic pattern of fly-tipping from our FOI sample had the same patterns as the fly-tipping statistics published by DEFRA, providing some confidence in the representativeness of the sample. The processed data are included in Additional file 1.

Potential sources of error in recorded fly-tipping data are similar to those for police recorded crime data (see Tseloni & Tilley, 2016). In particular, not all fly-tipping incidents are reported and, of those reported, not all are recorded. Quality control would vary by source: local authority fly-tip records are typically generated by a waste operative at the scene, or based on a report by a member of the public without specialist equipment. Likewise, duplicate records, errors in the recording of size, type and location of incident, and lack of standardisation of terms, are potential sources of error.

In the UK, responsibility for clearing illegal dumping lies with the land-owner which means there may be little incentive to report it to authorities in most instances. It also means that most fly-tip data examined here relates to public land. Aspects of recording practice are also known to differ between authorities. For example, there is known variation in how ‘side waste’ is recorded. ‘Side waste’ is that which is placed alongside a lawful waste repository but not in the correct manner, such as waste from a residential collection that exceeds the maximum quantity permitted. Side waste was recorded by some LAs as fly-tipping but not recorded at all by others. Within individual authorities there could be variation: local authorities that pay contractors to remove fly-tip waste have a monetary incentive to record fly-tipping more accurately, for example.

DEFRA suggest that comparison of numbers between local authorities is inadvisable because some are more proactive than others in finding illegal waste, either through their own efforts or by making it easier for the public to report it (DEFRA, 2019). However, with the simplifying assumption that recording practices in individual authorities remained relatively invariant,^5^ this does not preclude the type of trend analysis undertaken here.

### Analysis

Local authorities provided data in different formats, so the first step was to format for consistency. Some of the data covered shorter time periods than requested and this affected the number of cases available for two types of analysis (both described further below): the year-on-year comparison analysis required data for 2019 and 2020 whereas the ARIMA analysis required data for 2017 to 2020 inclusive. Table 2 shows the distribution of responses received by region and the number suitable for the analyses.

**Table 2:** Local authority responses by region and suitability for analysis

Region	Number of local authority’s responding		
	Monthly counts	Suitable for ARIMA analysis	Suitable for year-on-year analysis
South of England incl. London	73	50	55
English Midlands	53	43	46
The North of England	43	30	34
Scotland	29	25	25
Northern Ireland	5	4	4
Wales	13	9	9
Total	216	161	173

The general approach here is to compare the number of fly-tipping incidents in 2020 to the number expected in the absence of the pandemic. By comparing the observed and expected trends, the impact of the pandemic can be inferred. This is similar to the widely-used approach for estimating excess-deaths in the pandemic (Spiegelhalter & Masters, 2021) and for calculating expected crime rates (Ashby, 2020). The primary technique used here was Auto-Regressive Integrated Moving Average (ARIMA) time series modelling. Data from January 2017 to February 2019 inclusive was used to estimate the expected trend for 2020.

The ARIMA method adjusts for long-term trends and seasonal variation. After adjustment it uses the residual data to understand how data points that are close in time relate to each other. It includes ‘auto correlation analysis’ to capture how previous data points, typically within the last three units of time, affect the next data point. This procedure was followed because, with time series data, we cannot assume the monthly data to comprise independent samples from the same distribution (Hyndman & Athanasopoulos, 2018). Our ARIMA models were built using the auto arima function from the forecast package in R (Hyndman & Khandakar, 2008), as were the statistically significant bounds (assuming a normal distribution) that were calculated from the variation in the data.

Urban and rural differences were explored using the Office for National Statistics (ONS) area classifications for local authorities in England (ONS, 2016). The ONS classification had six categories which, for clarity and after confirming there was minimal loss of patterns, were aggregated into three for present purposes: Urban (comprising ‘Major Urban’), Semi-rural (including: ‘large urban’, ‘other urban’ and ‘significant-rural’) and Rural (including: ‘rural-50’ and ‘rural-80’).

## Results

### Number of fly-tipping incidents

**Fig. 1:**
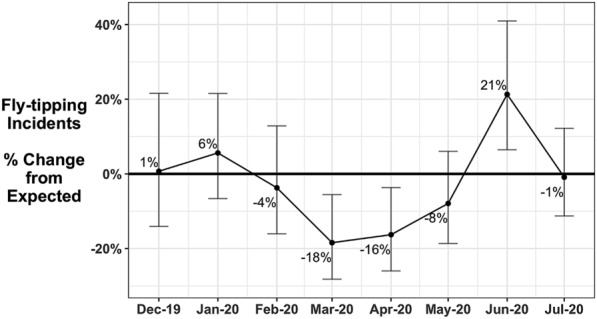
Percentage differences between expected and observed numbers of waste incidents (with drop-bars for 95% confidence intervals), December 2019–July 2020

During the first national lockdown, the number of illegal dumpings first declined significantly in March and April, then resurged significantly in June (Fig. 1). In March and April, the number of illegal dumping incidents was 18 then 16 percent below the expected monthly levels. In June, the illegal dumping was 21 percent above the expected level. In these 3 months the deficit or excess was statistically significantly different from expected, which is shown as those months where the drop-bars (showing the 95 percent confidence intervals based on past trends) do not cross zero. That is, the dumping deficit in March and April, and excess dumping in June, can reasonably be attributed to the lockdown policies.

**Fig. 2:**
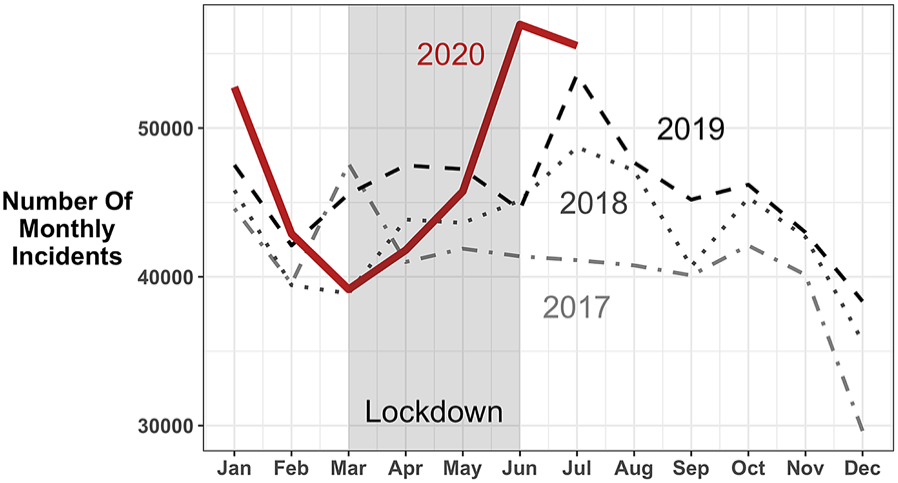
Monthly counts of recorded fly-tipping incidents for 2017, 2018, 2019 and January–July 2020

Figure 2 shows monthly counts in the number of illegal dumping incidents for all areas returning data for the full date range. It shows a general upward trend in incidents and some seasonal variation over winter (decreases in December and increases in January and the summer months). The ARIMA analysis (used for Fig. 1) accounted for these characteristics which means that we are confident that the differences found during the pandemic were not due to long-term or seasonal patterns in illegal dumping.

### Rural–urban differences

**Fig. 3:**
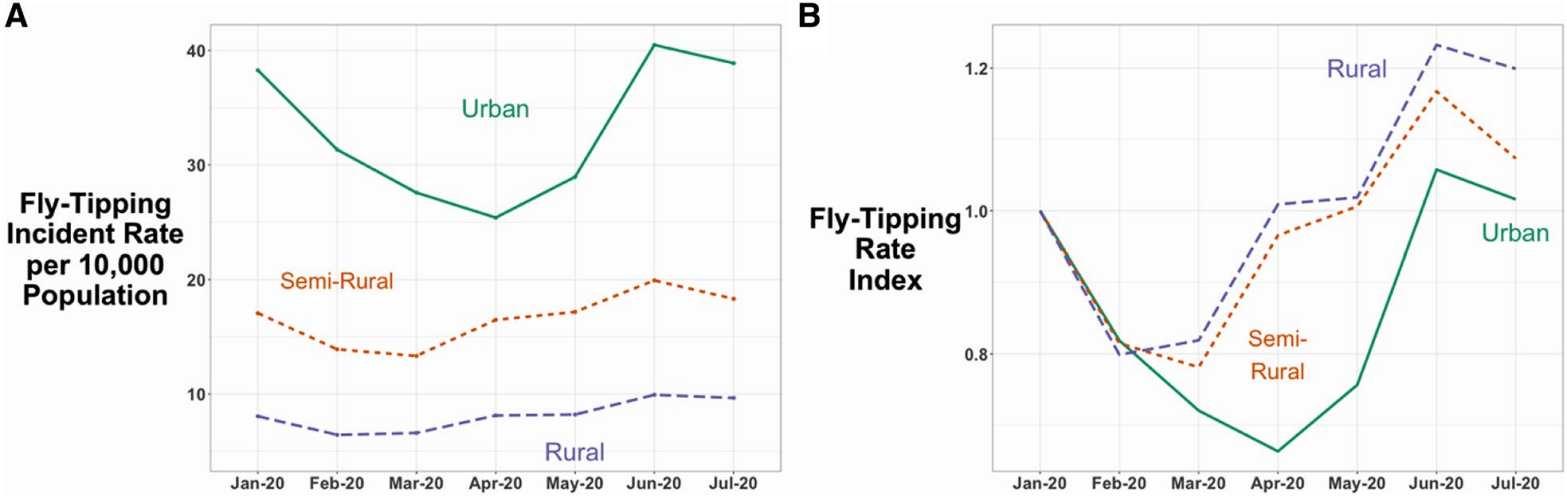
Changes in fly-tipping incidents in urban, semi-rural and rural areas, January–July 2020 (per 10,000 residents)

Figure 3 shows trends in illegal dumping incidents per 10,000 population for January to June 2020 for 36 urban, 82 semi-rural and 46 rural areas in England. Illegal dumping rates were sometimes four times higher in urban than rural areas. This suggests that the national trend may have been primarily due to change in urban areas with the more pronounced decline. However, the dumping deficits of the first national lockdown were not found in rural areas or in semi-rural/urban areas, travel to which was inhibited by movement restrictions.

Figure 3 suggests that the national picture was dominated by change in illegal dumping in urban areas. It also provides insight into why some rural areas may have appeared, as represented in the media, to have excess dumping during the first national lockdown. This issue is explored further below through closer examination of individual areas reporting excess dumping during the first national lockdown. Panel B is indexed to 1 in January 2020, and confirms that the early-lockdown decrease was concentrated in urban areas.

### Changes for individual areas

**Fig. 4:**
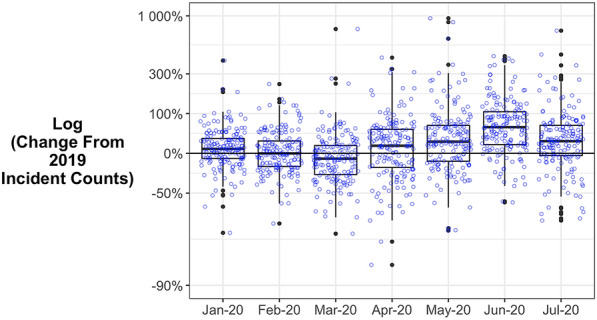
Boxplots of monthly changes in local authorities in 2020 relative to 2019

Although most areas followed the broad national trend, some experienced idiosyncratic changes. Figure 4 shows the change in each local authority relative to what was expected, using year-on-year logged changes in monthly incident counts (i.e. comparing each of the 7 months in 2020 to the same month in 2019). Each blue circle refers to an individual local authority. Boxes captures the interquartile range, the central line in each box is the median, and the whiskers (the vertical lines) show the range apart from outliers (the circles outside the whiskers). Note that the ordinate (Y-axis) is a log scale and that the percentage change in a few areas was large, particularly a small number of increases.

The distributions for January and February show there was considerable variability in the year-on-year change even before the pandemic. This means that large authority-level percentage changes during the pandemic are not necessarily a surprise: they occur routinely. Secondly, Fig. 4 shows that although the national picture was relatively clear—a dip early in the pandemic followed by a steady rise to June—there were isolated areas that experienced unusually large monthly changes. If the news reports highlighted in the introduction were focused on these outliers, then they may have been true at the local level while unrepresentative of the national picture. Note that the trend around zero in Fig. 4 is different to that of Fig. 1. This is because Fig. 4 is not weighted by number of incidents, and so does not reflect the findings that urban areas with the highest counts experienced the larger declines.

### The concentration of fly-tipping

**Fig. 5:**
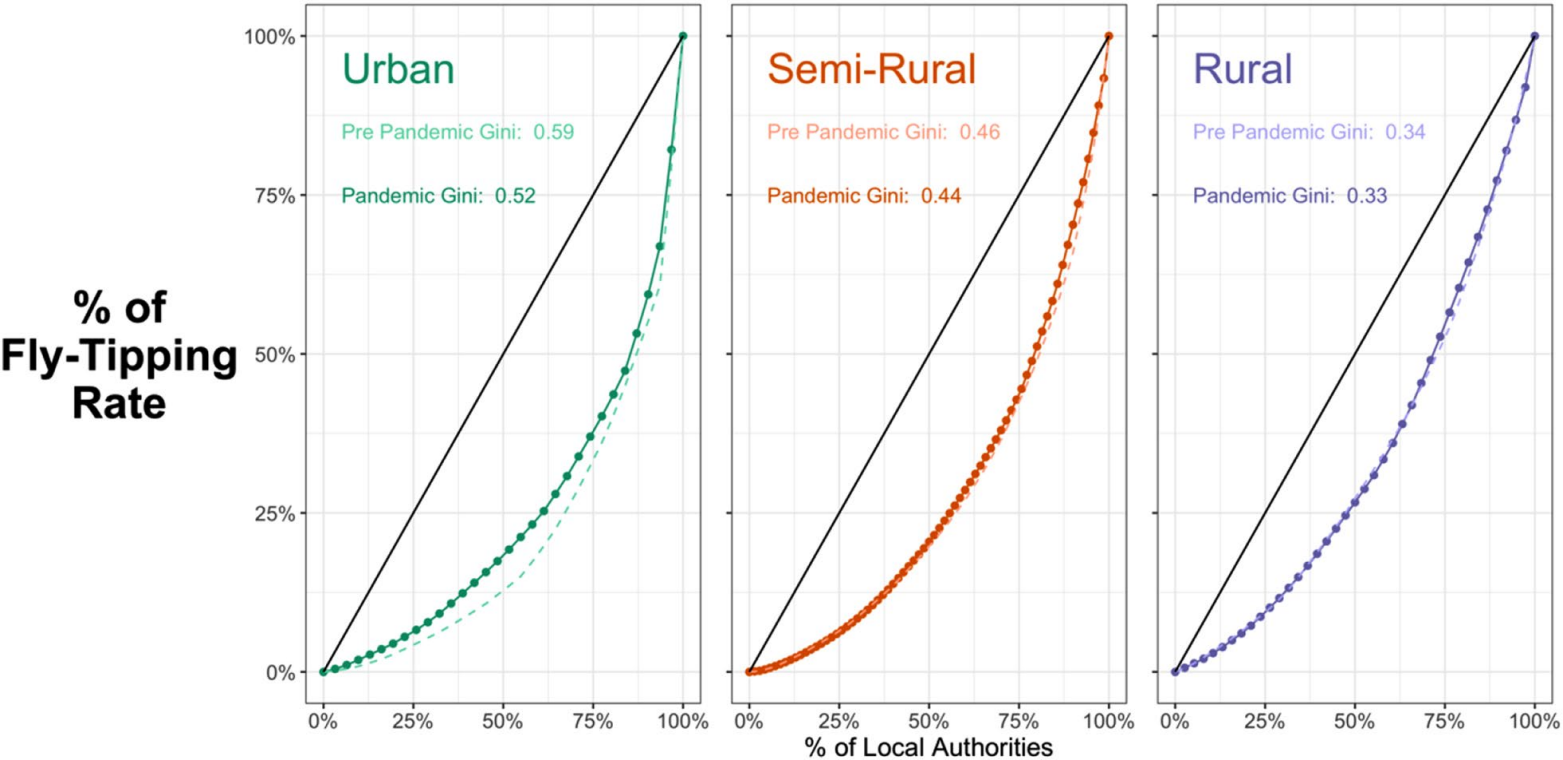
Concentration of fly-tipping by area type

Most crime-related phenomena are highly concentrated. This section sheds some initial light on the extent to which this is true for fly-tipping. Figure 5 shows Lorenz curves and Gini coefficients for fly-tipping rates aggregated for urban, semi-rural and rural areas. The black line depicts a line of equality (each local authority would have the same rate). The further the coloured line from the central line, the greater the inequality in the distribution of fly-tipping and the higher the corresponding Gini coefficient. A Gini coefficient of 0 would indicate that all areas experienced the same fly-tipping rates. A Gini coefficient of 1 would indicate that a single area experienced all the fly-tipping. The pre-pandemic data spans April to June 2019 and the pandemic data the same months in 2020.

Figure 5 indicates that fly-tipping is fairly concentrated within high-rate areas. Regardless of whether the area was urban or rural, those areas with a high rate accounted for a disproportionate amount of the fly-tipping. However, fly-tipping is more concentrated in urban than in rural areas. By this metric, in urban areas, 15 percent of urban local authorities account for 50 percent of urban fly-tipping incidents. However, levels of concentration changed little during this period of the lockdown.

Analysis of the number of incidents may overlook changes in the volumes of illegally dumped waste material. The above analysis was also conducted with counts converted to volume metrics where the data allowed. However, this analysis is not presented here because there were no significant changes from the findings relating to the number of incidents.

## Discussion

During April and May 2020 there was a statistically significant aggregate decline in recorded illegal waste dumping at the national level, relative to the expected level. This was contrary to expectation. However, within this aggregate picture there were a few isolated cases where major increases occurred.

Urban areas accounted for a disproportionate change in fly-tipping. Most shops, services, cafes, and other businesses which concentrate in urban areas, were closed during lockdown. This would have reduced the volume of business waste material needing disposal, meaning fewer rewards from fly-tipping. Most household-level collections of perishable food waste continued during lockdown, so households would not have needed to fly-tip such waste. In addition, it is reasonable to expect there was increased actual and perceived natural surveillance in more densely populated urban areas. Before the pandemic, fly-tipping activity was provided significant camouflage by the routine movements of daily life: other vehicles and people undertaking legitimate outdoor activities. In lockdown, this cover was largely removed. Moreover, the risk of being caught breaching lockdown laws would bring increased risk of detection of the more serious offence of fly-tipping. As a result, urban fly-tipping of non-perishable waste was particularly effected. It could wait.

Fly-tipping resurged when lockdown laws were relaxed, and normal business activity and everyday movement began to return. Both the volume of waste material produced in urban areas and the cover provided by lawful activities, increased. Any backlog of fly-tipping could be cleared under the new cover provided and the reduced risk of fly-tippers being stopped for breaching lockdown laws. Hence the resurgence is likely to be a form of temporal displacement as fly-tippers cleared the backlog.

Were the media reports misleading in reporting huge increases in fly-tipping? At the national level, they were certainly unrepresentative of what occurred. However, we also found a handful of areas where dramatic increases were experienced. If the media focused on these outliers, this would explain the discrepancy. This study provides evidence that fly-tipping declined early in the pandemic but then bounced back. This highlights the importance of rigorous empirical research on issues where the answers may initially appear “obvious”.

Do these findings apply to other times, places, and problems? Waste services can be disrupted for other reasons including strikes, severe weather events, and other disease outbreaks. The likelihood to which these affect illegal waste dumping will reflect the extent of disruption of services as well as the context: here, the broader lockdown laws were identified as highly influential, and these or similar are less likely for some other types of extreme events.

More broadly, compliance is an important issue for public policy. In addition to waste disposal, it applies to many issues including business compliance, tax payments, health and safety, and data protection. The present study raises questions, particularly where compliance mechanisms are disrupted. It suggests that relatively short-term disruptions to mechanisms that facilitate compliance do not necessarily trigger increases in offending. This suggests that further case studies of offending faced with disruption to opportunities and temptations for compliance to regulatory regimes would be a fruitful area of research.

It could be the case that waste was dumped with the same pattern during the pandemic as it was before, but it is a disruption to the reporting that is being observed in the data. We think this is unlikely. The most pronounced changes in fly-tipping were seen in the urban areas where people were generally confined and conducted their local exercise. In the rural areas where there were less allowable reasons for travel during the pandemic, the dip in fly-tipping was not as pronounced, which would not be consistent with a dearth then rapid influx of visitors to report the fly-tipping in rural areas. However, we acknowledge that a more detailed understanding of where the waste was tipped would provide a more definitive answer. Additionally, there is some evidence that a minority of councils ceased pro-active measures to find fly-tips during the lockdowns, though the extent of this is unclear and is offset by data from other LAs that show they were better able to respond to fly-tips, presumably because there were fewer incidents. A similar artifact was found in crime data where the increase in recorded drug crime was thought to be because of increased police availability as other crimes declined (Langfield et al., 2021).

Data limitations were discussed earlier. They include sample representativeness, the non-standardisation of local data collection, and the under-reporting of fly-tipping on private land.^6^ Few administrative data sets perfectly capture the phenomena they are intended to describe (on crime, for example, see Maguire & McVie, 2017, Tseloni & Tilley, 2016; on COVID-19 see Spiegelhalter & Masters, 2021; for general discussion see Becker, 2017). In the present study, data shortcomings were unlikely to mean that the overall results are misleading, but if waste crime is to be tackled effectively then improvements to data quality will improve future studies. Along these lines we make three recommendations. First, DEFRA should publish data with at least monthly (and preferable weekly or daily) granularity; publishing quarterly data is temporally insufficient for detailed analysis. In particular, monthly data would facilitate analysis of seasonal effects. Second, publishing geocoded data, even at the LSOA level, will facilitate more informed research, particularly with respect to the role of spatial clustering. Third, there is a need to measure fly-tipping on private land more accurately.

## Conclusion

Contrary to expectation, fly-tipping decreased statistically significantly below expected levels early in the pandemic. This is interpreted as an enhanced compliance effect. Most households do not usually fly-tip and did not begin, and most habitual fly-tippers did not breach lockdown law to dump non-perishable waste. The perceived risk of illegal dumping would be high during lockdown, more so in densely populated urban areas where public movement  - a pre-requisite for fly-tipping - was conspicuous. Once lockdown restrictions were eased and higher levels of public movement returned, fly-tipping increased significantly above expected levels. We interpret this as a form of temporal displacement, as fly-tippers overcame their backlog, making up for lost crime.

There is a clear need for improvements in data collection relating to fly-tipping. Many of the problems of the currently DEFRA-collected data were discussed earlier. The data that is currently made publicly available allows little detailed analysis of events, seasonal or other patterns, and does not facilitate identification of hot-spots or other forms of concentration.

## Supplementary Information

**Additional file 1.** Monthly fly-tipping counts by month and local authority

## Data Availability

The cleaned count data is included in Additional file.

## Footnotes

1 There are other forms of illegal waste disposal that are important but lie beyond the scope of this paper, such as transnational traffic in waste and corporate pollution practices of various kinds.

2 There are variations between England, Scotland, Wales, Northern Ireland and Scotland. There are also variations in practice across areas within each country. For the purposes on this paper, these details are ignored.

3 intermediaries is used instead of ‘guardians’ since the former includes intimate handlers (orientated to potential offenders) as well as guardians (orientated to potential targets).

4 https://www.gov.uk/make-a-freedom-of-information-request/how-to-make-an-foi-request.

5 A reviewer suggested that waste disposal staff absences due to COVID-19 may make this assumption implausible. However, none of the staff that we spoke to, predominantly in June 2020, noted this issue. More definitive evidence can be inferred from NHS clinical staff absence rates at the time, which were relatively low: For clinical staff the absence rates attributable to covid were 0.9%, 2.1%, 0.9% and 0.4% respectively NHS (2020). NHS staff are on the frontline against covid, and we can assume that waste disposal staff absence rates could be equivalent, but most likely will have been lower. In the early stages of the pandemic, covid had just not spread around the country that fast, so not that many people were sick with it.

6 While fly-tipping on private land is the responsibility of the land-owner, some finds its way into local authority records. This was the case in the data collected for this study, as it is in DEFRA’s reports which, for 2020/21 included around 18,000 fly-tips on private land and 6000 on commercial land.
